# Dr. Mouat on the Bengal Medical Returns

**Published:** 1847-04

**Authors:** 


### Art. IX.

*Observations on the Nosological Arrangement of the Bengal Medical Returns; with a few cursory Remarks on Medical Topography and Military Hygiene.* By Fred. J. Mouat, m.d., Assistant Surgeon, Bengal Army, Professor of Materia Medica and Medical Jurisprudence in the Bengal Medical College.—*Calcutta*, 1845. 8vo, pp. 64.

Having been long deeply impressed with the advantages to be derived from the application to medicine of those strict rules of observation under which the exact sciences have made such rapid progress, we hail with pleasure the indications of an increasing attention on the part of the profession to that desirable object. The pamphlet now before us gives us good grounds to hope that the wide field of investigation, presented by our Indian possessions, is about to be cultivated with that zeal, and in that manner, which its extent and importance justly claim for it, and that a race is springing up who will turn to good account the magnificent opportunities they possess of studying the influence of climate upon, and the progress of disease among various races. An old author observes that "it is no shame not to know that which one has not had an opportunity of learning, but it is scandalous to profess knowledge and remain ignorant," and this remark may be justly extended to those who have opportunities and neglect them.

When the very able statistical Returns of the Army were presented to Parliament, Mr. Hume moved in the House of Commons for the production of similar returns from the army of the Hon. East India Company, which were ordered, but it was found on investigation, that no documents existed which were calculated to furnish materials for constructing comprehensive statistical tables. To remedy this for the future, and remove the stigma which cannot but attach to them for having so long neglected the opportunity of collecting materials for the advancement of medical science, the Medical Board in Bengal issued a new form of returns which we must say is the most artificial and *unpractical* (if we may coin a word) we have ever seen. The object of the present pamphlet is to point out some of the imperfections which attach to them.

In the statistical reports which have been already published in this country, three different nosological arrangements have been adopted; the navy having followed that of Cullen, while the army and the registrar-general framed systems for themselves. We presume that Cullen's classification was followed by the navy authorities, simply because it was at the time of its adoption the best in common use: every one knows that it is a very defective one. Diseases are brought together which have no affinity to each other, either as regards their seat, or the causes likely to produce them. For example we find ophthalmia in the same class with lumbago, phthisis with hæmaturia, syphilis with icterus, aneurism with hernia humoralis, luxatio with hernia and prolapsus ani, &c.

In the military statistical reports, the arrangement of Cullen, which was used in the returns, was abandoned, and one of a more practical nature adopted. If we may judge by the benefits derived from the adoption of the suggestions founded on the results of these reports, the classification appears well suited to the investigation of the diseases of bodies of men at the period of life during which soldiers usually serve.* The statistical nosology followed in the reports of the registrar-general, is well adapted for the returns of a general population, but there are many peculiarities in the condition of the soldier which would lead us in any inquiry into the health of this class to prefer that employed in the military reports.

The classification adopted in the new forms issued by the Medical Board in Bengal, appears to be that of Dr. Mason Good. The diseases are divided into seven classes, viz. those of the digestive, respiratory, sanguineous, nervous, sexual, and excernent functions, and those from external violence. We will not stop to inquire with Dr. Mouat, what is meant by sanguineous *function,* digestive *function,* &c., but briefly notice a few of the objections to the classification.

The only disease under the head of "the respiratory function" is asthma. "This is an excellent illustration," says Dr. Mouat, "of the difficulty, if not impossibility, in the present state of our knowledge, of classifying diseases by any physiological arrangement such as that attempted in this table. It has a *primâ facie* appearance of philosophic accuracy, which on closer investigation will not stand the test of rigid analysis, and is not found to answer any beneficial purpose." After adverting to the various theories regarding the nature of asthma, he adds, "no conjectures or theoretical speculations are hazarded in placing it under the head of diseases of the respiratory organs. For the same reason, catarrh, pneumonia, pleuritis, bronchitis, and phthisis, ought to have been placed in the same order, as affecting this system,"—an opinion in which every practical man will probably concur.

We cannot afford space to follow our author through the remarks on the various classes, but we think it will be admitted that, for practical purposes, the classification must be very deficient which includes in the same category, fevers, ophthalmia, rheumatism, phthisis, syphilis, and ulcers; which associates cataract with delirium tremens, and psora with

* For the arrangement adopted and the practical advantages which have arisen out of these Reports, see British and Foreign Medical Review for April, 1844, p. 313.

dropsies; and which exhibits gonorrhea as the only disease of the "sexual function!" We must also join in protesting against the use of the term "alii morbi;" whatever classification may be adopted, medical officers should be imperatively required to return every case of disease under its proper head. The lumping of several diseases in this way, diminishes materially the value of the returns, and is at best but a poor economy of stationery.

It is most justly remarked by our author, that

> "It cannot fail to be a source of regret that the system of returns required from the Indian army, should not be identical with that adopted in Her Majesty's service: the value of such uniformity in extending our knowledge of the medical economy of troops serving in every quarter of the extended empire subject to Great Britain; of the closer approximation to truth that would result from calculations spread over so wide a field, and deduced from numbers that would constantly tend to diminish the amount of error arising from local and accidental causes in smaller bodies of men, is almost self-evident to any one who has studied attentively the important subject of statistics."

The advantages to be derived from this uniformity in the returns have been brought under the notice of the authorities since Dr. Mouat's paper was written, and we trust will be duly considered in the arrangements which are to be made in India for collecting military statistics.

Dr. Mouat has appended to his paper, a brief analysis of the various works that have been published on the medical topography of some of the stations in the Bengal Presidency, which may prove useful to those desirous of ascertaining what information is extant on this subject. In noticing one of these, he quaintly observes "much valuable matter and careful registers of the hygiène of prisoners may possibly exist in the records of Government, but as they have never been published or made known, the information is of no practical use."

We are at all times happy to have it in our power to notice the labours of our brethren in India, because we consider them entitled to much more credit for their exertions under all the disadvantages and discouragements to which they are exposed, than we are who enjoy the bracing air of a temperate clime. We believe there is no want of zeal on the part of the officers of the Indian medical department, but that they are anxious to promote the cause of science, and contribute their quota to the advancement of their profession; and we trust the authorities will by liberal arrangements soon enable them to produce a series of statistical returns worthy of being ranked with the best works of this nature. Dr. Mouat, who is but young in the profession, is entitled to great praise for the able and zealous manner in which he has endeavoured to direct the attention of the ruling powers to the important subject treated of in his pamphlet.

